# Supplementary material for: Calpains Orchestrate Secretion of Annexin-containing Microvesicles during Membrane Repair
Source: bioRxiv. 2024 Sep 6:2024.09.05.611512. Preprint. [Version 1] doi: 10.1101/2024.09.05.611512 (PMC11398502; doi:10.1101/2024.09.05.611512)
Supplement: Supplement 5 [file NIHPP2024.09.05.611512v1-supplement-5.pdf]

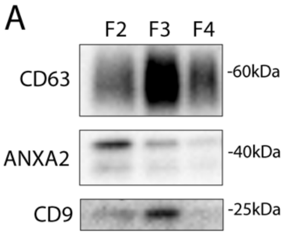

S1. Annexin-containing extracellular vesicles are distinct from exosomes.

Immunoblots show distribution of EV markers across a sucrose step gradient of the conditioned medium 100k × g pellet fraction. Samples taken from low density (F2-Fraction #2) to high density (F4-Fraction #4).

bioRxiv preprint doi: https://doi.org/10.1101/2024.09.05.611512; this version posted September 7, 2024. The copyright holder for this preprint (which was not certified by peer review) is the author/funder, who has granted bioRxiv a license to display the preprint in perpetuity. It is made available under aCC-BY 4.0 International license.

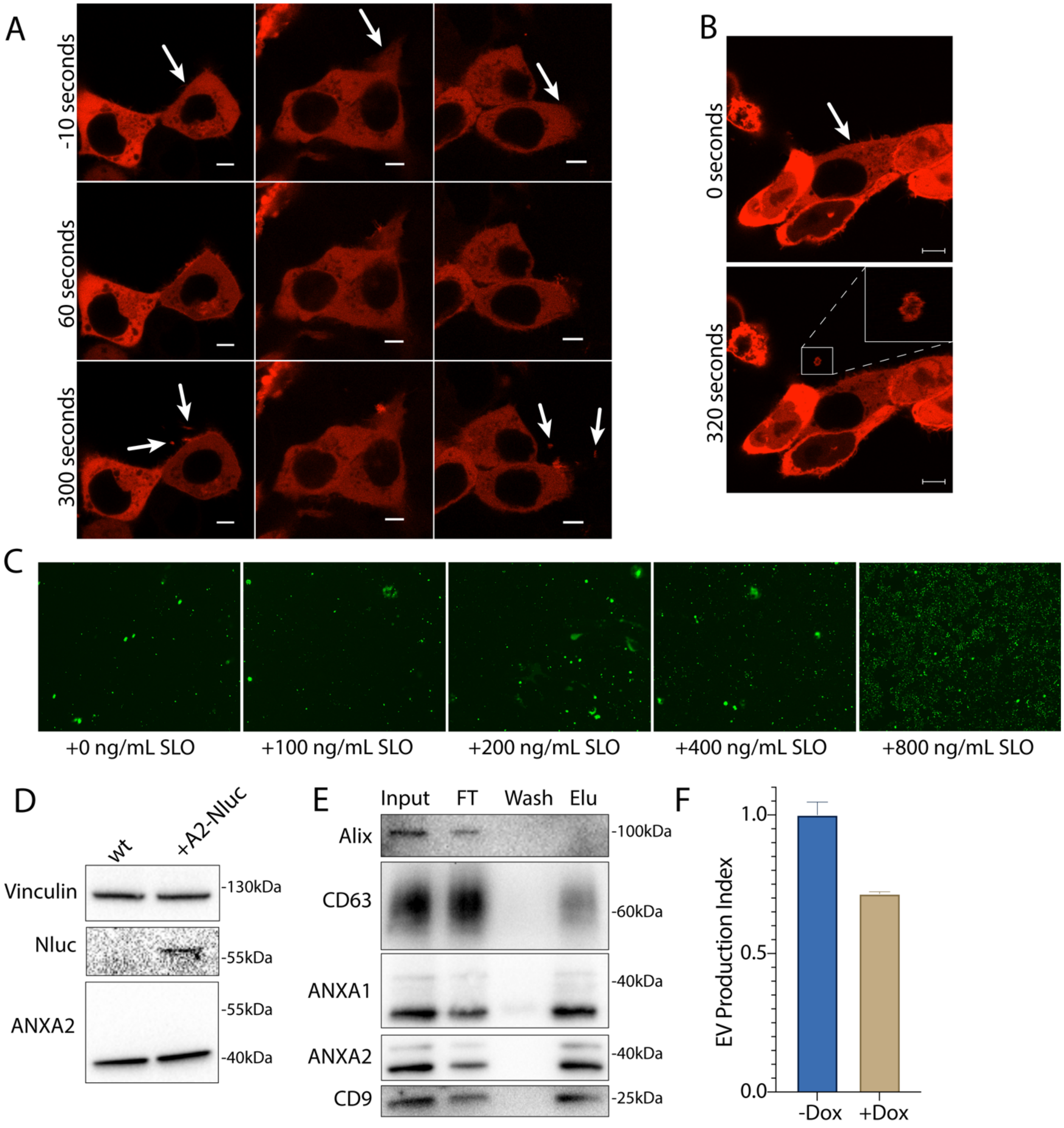

S2. Annexin-containing extracellular vesicles are shed from the repair scab after plasma membrane damage.

(A) Confocal micrographs of ANXA2-mScarlet recruitment in three laser ablation experiments are shown. Image times are relative to the first image taken post ablation. White arrows in the top panes indicate the sites of ablation. Arrows in the bottom panes indicate extracellular vesicles. Scale Bars: 5 µm. (B) Representative confocal micrographs of ANXA2-mScarlet shedding are shown. Image times are relative to the first image taken post ablation. White arrows in pane I indicate the site of ablation. Scale Bars: 5 µm. (C) Representative widefield micrographs of cells stained with 1 µM Sytox Green after a treatment period with the indicated SLO concentration and recovery period. (D) Immunoblots show expression of annexin A2-Nluc

bioRxiv preprint doi: https://doi.org/10.1101/2024.09.05.611512; this version posted September 7, 2024. The copyright holder for this preprint (which was not certified by peer review) is the author/funder, who has granted bioRxiv a license to display the preprint in perpetuity. It is made available under aCC-BY 4.0 International license.

(A2-Nluc) using a low expression promoter. (E) Immunoblots show enrichment of EV markers after capture with immobilized annexin A5 from the conditioned medium 100k × g pellet fraction (FT-Flow Through, Elu-Elution). (F) EV production index from ANXA2-Nluc cells expressing mCherry-VPS4a (dominant mutant) under control of a doxycycline-inducible promoter. Cells were pretreated with 200ng/ml doxycycline (Dox) or DMSO for 6 h followed by treatment with 200 ng/ml SLO.

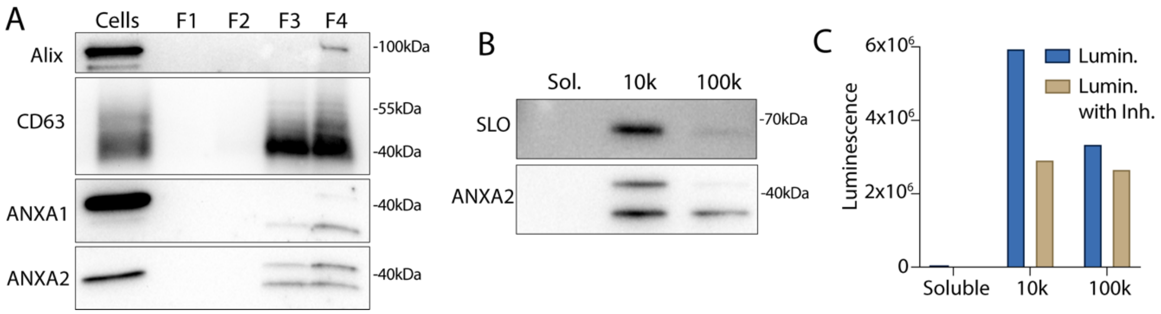

S3. Annexins within extracellular vesicles are shifted in apparent molecular weight.
(A) Immunoblots show enrichment of indicated EV markers relative to cell lysate after treatment of C2C12 myotubes with 200 ng/mL SLO. F1, F2, F3, and F4 refer to buoyant fractions of a sucrose step gradient of the conditioned medium 100k × g pellet fraction, moving from low to high density. (B) Immunoblot and (C) luminescence analysis of the 10k × g pellet fraction

bioRxiv preprint doi: https://doi.org/10.1101/2024.09.05.611512; this version posted September 7, 2024. The copyright holder for this preprint (which was not certified by peer review) is the author/funder, who has granted bioRxiv a license to display the preprint in perpetuity. It is made available under aCC-BY 4.0 International license.

(10k), the 100k × g pellet fraction (100k), and the remaining soluble supernatant (Sol.) after serial centrifugation of conditioned media from ANXA2-Nluc cells treated with 200 ng/ml SLO. For each fraction, Nluc luminescence (lumin.) was measured with or without membrane impermeable Nluc inhibitor (Inh.).

bioRxiv preprint doi: https://doi.org/10.1101/2024.09.05.611512; this version posted September 7, 2024. The copyright holder for this preprint (which was not certified by peer review) is the author/funder, who has granted bioRxiv a license to display the preprint in perpetuity. It is made available under aCC-BY 4.0 International license.

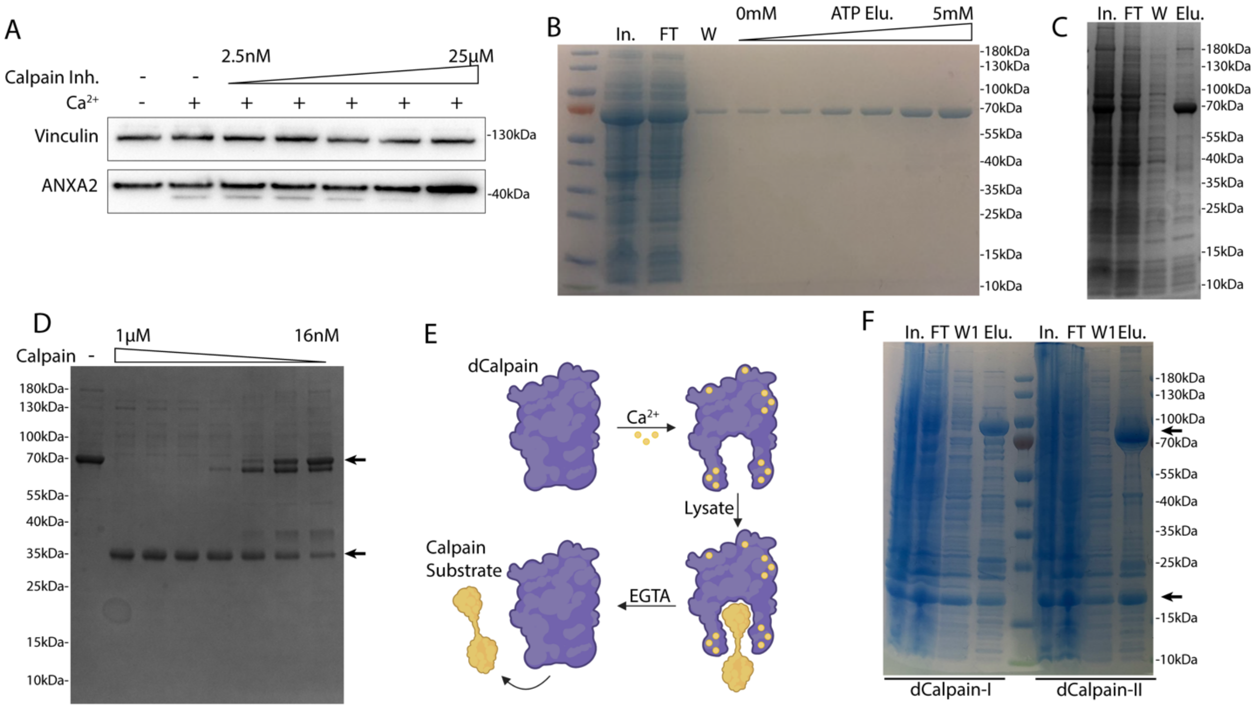

S4. Calpain-1/2 cleave annexins which are then shed in microvesicles.

bioRxiv preprint doi: https://doi.org/10.1101/2024.09.05.611512; this version posted September 7, 2024. The copyright holder for this preprint (which was not certified by peer review) is the author/funder, who has granted bioRxiv a license to display the preprint in perpetuity. It is made available under aCC-BY 4.0 International license.

(A) Immunoblot analysis of cytosol fractions after incubation with or without 1 mM Ca^{2+}, with a range of concentrations of ALLN inhibitor (Calpain Inh.). (B) Coomassie-stained gel showing purification of annexin A2-Halo from *E. coli* (In- lysate input, FT- 100k x g pellet fraction flow through, W- $CaCl_2$-containing 100k x g pellet wash, ATP Elu- elution from 100k x g pellet fraction with increasing concentrations of ATP). (C) Coomassie-stained gel showing purification of annexin A6-HA from *E. coli* (In- lysate input, FT- 100k x g pellet flow through, W- $CaCl_2$-containing 100k x g pellet wash, Elu- elution off 100k x g pellet with 10mM ATP). (D) Coomassie-stained gel showing the mobility of recombinant annexin A6-HA, incubated with a range of concentrations of purified, porcine calpain-1. Arrows indicate uncleaved and cleaved product. (E) Schematic illustrating substrate binding and elution of substrates (yellow) to catalytic cysteine-to-serine mutant calpain baits (dCAPN, purple). (F) Coomassie-stained gel showing initial, his-tag purification of CAPN1[C115S]-3xFlag-6xHis (dCalpain-I) and CAPN2[C105S]-3xFlag-6xHis (dCalpain-II) in complex with CAPNS1(86-268) from *E. coli* (In- lysate input, FT- bead flow through, W- bead wash, Elu- elution from Ni^{2+} with 300 mM imidazole). Arrows indicate calpain proteins.

bioRxiv preprint doi: https://doi.org/10.1101/2024.09.05.611512; this version posted September 7, 2024. The copyright holder for this preprint (which was not certified by peer review) is the author/funder, who has granted bioRxiv a license to display the preprint in perpetuity. It is made available under aCC-BY 4.0 International license.

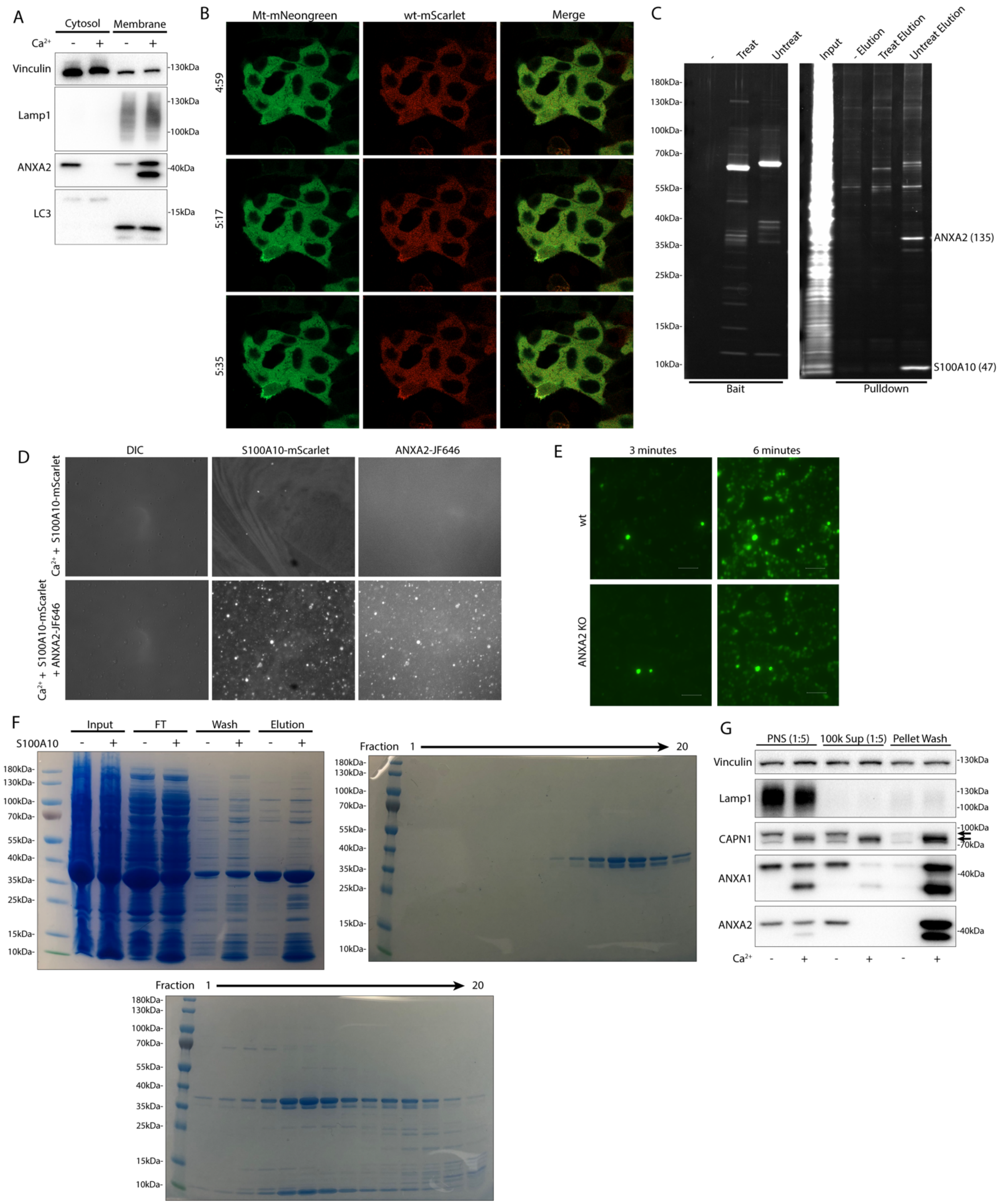

S5. Calpain cleavage attenuates the membrane binding and scabbing activity of annexin A2. (A) Immunoblot analysis of cytosol and membrane factions, with or without 1 mM Ca^{2+} added prior to fractionation. (B) Representative confocal micrographs of ANXA2-mScarlet (wt-mScarlet) or ANXA2[P27D, P28D]-mNeonGreen (Mt-mScarlet)-expressing cells. Image times are

bioRxiv preprint doi: https://doi.org/10.1101/2024.09.05.611512; this version posted September 7, 2024. The copyright holder for this preprint (which was not certified by peer review) is the author/funder, who has granted bioRxiv a license to display the preprint in perpetuity. It is made available under aCC-BY 4.0 International license.

(A) EV production index from ANXA2-Nluc cells treated with the indicated combinations of 200ng/ml SLO and 20 µM calpain Inhibitor, ALLN. (B) Immunoblot analysis of lysates from wildtype cells (wt), annexin A2 knockout cells (KO), and annexin A2 knockout cells expressing wildtype annexin A2-Nluc (KO + wt) or ANXA2[P27D, P28D]-Nluc (KO + Mt). (C) EV production index from wildtype ANXA2-Nluc (wt) or ANXA2[P27D, P28D]-Nluc (Mt) cells treated with or without SLO. (D) Quantification over time of the repair scab intensity from FM1-43 stained (2.5 µM) annexin A2 knockout cells rescued with ANXA2-mScarlet (wt) or ANXA2[P27D, P28D]-mScarlet (Mt). Cells (6) were ablated for each condition. (E) Confocal micrographs of 8 laser ablated annexin A2 knockout cells rescued with either wild-type ANXA2-mScarlet (wt) or ANXA2[P27D, P28D]-mScarlet (Mt). Images are 2 min, 30 sec after ablation. Scale Bars: 5 µm. (F) Schematic depicting the current model of plasma membrane repair and annexin^{+} MV secretion.
